# Respiratory sound analysis in the era of evidence-based medicine and the world of medicine 2.0

**Published:** 2018

**Authors:** E Andrès, R Gass, A Charloux, C Brandt, A Hentzler

**Affiliations:** * Department of Internal Medicine, Clinique Médicale B, Hôpitaux Universitaires de Strasbourg, Strasbourg, France; ** Technical Academy Fellow, Alcatel-Lucent, Independent expert, Bolsenheim, France; *** Department of Physiology and Lung Function Exploration, Hôpitaux Universitaires de Strasbourg, Strasbourg, France; **** Department of Cardiology, Clinique Médicale B, Hôpitaux Universitaires de Strasbourg, Strasbourg, France; ***** Physics Engineer, General Director INCOTEC, Illkirch Graffenstaden, France

**Keywords:** State of the art, auscultation, respiratory sounds, lung sounds, crackles, wheezes, respiratory phase detection, spectral analysis, wavelet, respiratory phase classification, signal processing, artificial neural networks, genetic algorithm, multilayer perceptron, fuzzy rule-based identification system, rhonchus, snoring, squawk, stridor

## Abstract

Objective: This paper describes the state of the art, scientific publications, and ongoing research related to the methods of analysis of respiratory sounds.

Methods and Material: Narrative review of the current medical and technological literature using Pubmed and personal experience.

Results: We outline the various techniques that are currently being used to collect auscultation sounds and provide a physical description of known pathological sounds for which automatic detection tools have been developed. Modern tools are based on artificial intelligence and techniques such as artificial neural networks, fuzzy systems, and genetic algorithms.

Conclusion: The next step will consist of finding new markers to increase the efficiency of decision-aiding algorithms and tools.

## Introduction

Distinguishing between normal respiratory (lung) sounds and abnormal ones (such as crackles and wheezes) is crucial for establishing an accurate medical diagnosis. Respiratory sounds include all the invaluable information concerning the physiology and pathology of lung and airway obstruction [**1**].

Evaluation of the sounds produced by the human body can be traced back as far as ancient Egypt [**1**]. Papyrus records from the 17^th^ century B.C. have been uncovered describing listening to sounds inside the body as a way to learn about illnesses. Up to the beginning of the 19^th^ century, doctors still examined their patients this way, pressing the ear to the thorax to listen to the noises within [**2**]. We call this “immediate auscultation”.

**Fig. 1:**
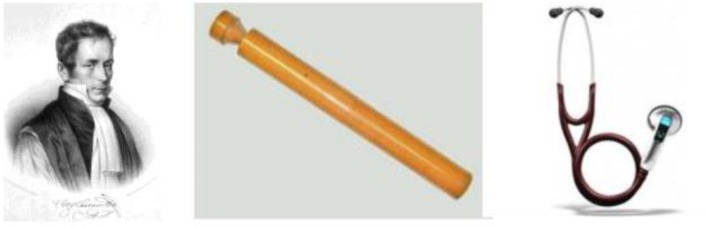
1a: Doctor René-Théophile-Hyacinthe Laennec, inventor of the stethoscope. 1b: The stethoscope is a system of transmission and amplification of sound by resonance. Its principle is rather simple. Using a specific interface (diaphragm or bell), a sound is picked up and then transmitted over a small distance to the user’s ears. Traditionally, the sound is transmitted in an aerial way via a conduit. 1c: The latest generation of stethoscopes is electronic and uses a microphone system and speakers to restore sounds (*adapted from the chapter: Advances and Perspectives in the Field of Auscultation, with a Special Focus on the Contribution of New Intelligent Communicating Stethoscope Systems in Clinical Practice, in Teaching and Telemedicine. InTech 2012.* *http://dx.doi.org/10.5772/48402*).

In 1817, Laennec created a new technique he labeled “*auscultation médiate*”, meaning auscultation through a medium (*Traité de l’auscultation médiate*, Paris, 1817) (**Fig. 1a**) [**1**]. Doctor Laennec also built the first paper-based (a cone made out of 24 sheets of paper) and wood-based stethoscopes (**Fig. 1b**). Not only did this enable him to listen to internal noises without being in direct contact with his patient, but it also provided a much stronger and clearer perception of the noises.

Since the 1800s, the stethoscope has become increasingly popular, having been adopted as the physician's primary medical tool. Before the turn of the century, this device looked much the same as it does today, with a binaural design, flexible tubing, and a rigid diaphragm. Bowles and Sprague developed the combined bell and diaphragm design in 1925, then shortly following World War II, Sprague, Rappaport, and Groom experimented with the design before finding the optimal combination of the classic double-tube Rappaport-Sprague stethoscope [**1**]. During the last decade, considerable progress has been made to improve the stethoscope (**Fig. 1c**) [**3**][**4**]. There is limited information on what the standardized use of a stethoscope for chest auscultation should be, due to the inherent inter-listener variability. Electronic auscultation and the automated classification of recorded lung sounds may help overcome some of these shortcomings. Innovative technologies that could project the stethoscope and auscultation approaches into the era of evidence-based medicine and the world of medicine 2.0 have emerged [**3**]. In fact, during the last decade, the Internet has become increasingly popular and is now an important part of our daily life. When new “Web 2.0” technologies are used in health care, the terms “Health 2.0" or "Medicine 2.0” may be used.

Our study describes the state of the art, scientific publications, and ongoing research related to the methods of analysis of respiratory sounds.

## Systematic review method

Two investigators (EA, RG) conducted independent literary research using *Medline* and *Google Scholar* up to January 31, 2018. Additional data was obtained from the references of identified studies, the *Cochrane Library*, and the *ISI Web of Knowledge*. We searched for studies relating to respiratory sound analysis. Keywords included “respiratory sounds”, “lung sounds”, “lung auscultation”, “electronic auscultation”, “acoustic signal processing”, “computerized respiratory sound analysis”, “computerized lung sound analysis”, “automated classification of respiratory sounds”, “automated classification of lung sounds”, “crackle detection”, “wheeze detection”, and “telemedicine 2.0”. We restricted our search to the English and French languages because both investigators were fluent in these two languages.

We independently reviewed and compared the resulting list of relevant articles and determined the eligibility of the full report and evaluated each of the included studies for their analysis of respiratory sounds. All the studies were analyzed by all the contributing authors.

## Physiological data

**Limits of human audition**

Studies were performed in order to test the human ear’s capability to detect crackles in auscultation signals **2**. The methods used consisted of simulated crackles superimposed on real breathing sounds. The results indicated that the most significant detection errors are owed to the following factors: – The intensity of the respiratory signal: deep breathing masks more crackles than superficial breathing; – The type of crackles: fine crackles are easily recognizable as their waveform differs more significantly from that of classic lung sounds; – The amplitude of the crackles.

It can be inferred from these studies that auscultation should not be used as the sole reference for validating automatic crackle detection algorithms.

Despite this, we still have some way to go before we perfectly understand the mechanisms linked to the creation of breath sounds. By recording and analyzing respiratory sounds, we will be able to improve this understanding [**3**] and establish an objective relationship between abnormal respiratory sounds and respiratory pathology. Furthermore, an objective analysis would enable classification systems to be developed [**1**][**4**] making it possible to precisely differentiate between normal and adventitious respiratory sounds.

While conventional stethoscope auscultation is subjective and difficult to reproduce from case to case, these systems should provide rapid, objective diagnostic help, with better sensitivity and reproducibility of the results [**5**]. Moreover, applications like diagnosis evaluation, monitoring, and data exchange through the internet are natural complementary tools to objective and automatic auscultation sound analysis. Sensory devices will enable long-term monitoring of patients at home or at the hospital. This could also be a useful solution for less-developed countries and remote communities [**6**]6. In addition, this type of system has the great advantage of keeping auscultation non-invasive and affordable. Sestini’s studies [**7**] indicate that an association between acoustical signal and image is beneficial to breath sound learning and understanding for medical science students.

**Mechanisms of breath sound production**

Sarkar [**8**] offers a useful description of breath sound production. Normal breath sound is produced by the air flow through the trachea-bronchial tree. However, not all types of airflow produce breath sounds. Only turbulent and vorticose airflow generates breath sounds. Airflow moves parallel to the walls in a parabolic shape as the air in the central layers moves faster compared to the peripheral layers, with little or no transverse flow. Thus, there is little mixing or collision between gas layers. Laminar flow pattern follows the Poiseuille equation.

**Propagation of respiratory sounds**

The propagation and deformation of breath sounds are linked to several factors [**9**]: – The acoustical response of the stethoscope, the asymmetry of the sounds (potentially indicating the presence of a pathology), the heterogeneous composition of the body (bones, muscles, and skin) that behave like filters; – The analysis point: measurements indicate that lung sounds are lower in amplitude than tracheal sounds.

**Definition of experimental conditions**

Rossi *et al*. [**10**] give recommendations concerning the experimental conditions required for recording respiratory sounds. They describe the optimal conditions (principally concerning background noise, including non-breathing sounds such as vocal sounds) and specific procedures according to the type of sounds targeted for recording (breaths, coughs, snores), information for the recording (diagnosis, evaluation of a therapy, monitoring), the age of the subject (baby, infant, child, adult), and the recording method (free field, endobronchial microphone).

In practice, for short recordings, a sitting position is recommended, while the patient should preferably be lying down for long recordings [**8**].

## Definition of terms

Nowadays, there are several definitions of common marker characteristics, such as wheezes and crackles [**1**]. Thus, a universal semantic must be created. Several works have tried to collect term definitions in relation to respiratory sounds and have created a definitive collection of 162 terms commonly utilized in the “*Computer Respiratory Sound Analysis*” (CORSA) [**5**][**9**][**11**].

Nevertheless, this still does not provide physicians with common definitions of the terms that are used. Consequently, the descriptions of sound characteristics are still based on imaged illustrations. For example, a wheeze is currently associated with a “whistling sound” and a crackle with “a sound of rice in a frying pan”.

Sovijarvi *et al.* [**1**] [**11**] provided accurate definitions of currently used terms in the pulmonary auscultation and sound analysis fields. We list the most pertinent below.

**Sounds**

*Adventitious sounds*: These are defined as additional respiratory sounds overlying normal breath sounds [**1**] [**11**]. They can be continuous (like wheezes) or discontinuous (such as crackles), and some can be both (like squawks). The presence of such sounds usually indicates a pulmonary disorder.

*Breath sounds*: These include normal and adventitious sounds recorded over the chest wall, trachea or at the level of the mouth [**1**] [**9**] [**11**]. They are created by airflow in the respiratory tract. Acoustically, these sounds are characterized by broad-spectrum noises at frequencies ranging according to the location they are detected in.

*Lung sounds*: These are all respiratory sounds heard or detected over the chest wall or within the chest, including breath sounds and adventitious sounds detected in this location [**1**] [**9**] [**11**].

*Normal breath sounds*: At the chest wall, a respiratory sound is characterized as low noise during inspiration and hardly audible during expiration [**1**] [**11**]. In the trachea, normal respiratory sounds are characterized by a broader spectrum of noises (containing higher-frequency components, for example), audible both during inspiratory and expiratory phases. Normal breath sounds include vesicular sounds, bronchovesicular sounds, bronchial and tracheal sounds, together with sounds emitted through the mouth.

*Abnormal sounds*: As opposed to those classified as “normal”, abnormal sounds are those which may indicate a lung problem, the absence of sound where there should be one or sounds detected where they should not ordinarily exist [**1**] [**9**] [**11**]. These usually indicate a lung problem, such as inflammation or an obstruction.

**Known trackers**

*Crackles*: These explosive and discontinuous adventitious sounds generally appear during inspiration [**1**] [**5**] [**9**] [**11**]. They are characterized by their specific waveform, duration and location in the respiratory cycle. A crackle can be characterized as fine (short duration) or coarse (long duration). Crackles usually indicate that there is a pathological process in the pulmonary tissue or airways. “Coarse” crackles occurring during the beginning of inhalation indicate a chronic bronchial disease. When occurring in the middle of inhalation they indicate bronchiectasis and when at the end of inhalation, they are generated by the peripheral bronchi and could be a sign of pneumonia. “Fine” crackles are generated by the peripheral bronchi. They are symptoms of infection or pulmonary edema. “Coarse” crackles sound like salt poured into a hot pan, while “fine” crackles sound more like Velcro strips being slowly pulled apart or a bottle of sparkling water being opened.

*Cough sounds*: These are transient sounds induced by the cough reflex with a frequency range of 50 to 3,000 Hz [**5**] [**11**]. The characteristics of cough sounds differ among pulmonary diseases. Cough sounds containing wheezes are typical in asthma.

*Rhonchus*: Rhonchus is a low-pitched wheeze containing rapidly damping periodic waveforms with durations >100 ms and a frequency <300 Hz [**1**] [**5**] [**11**]. Rhonchi can be found in patients with secretions, a narrowing of the central airways or presenting increased airway collapsibility or chronic bronchitis.

*Snoring sounds*: These are low-frequency respiratory sounds with periodic components (fundamental frequency: 30-250 Hz), usually detected during sleep and caused by abnormal vibrations in the walls of the oropharynx [**1**] [**9**] [**11**]. While typically it is a sound of inspiration, some small snoring sounds may appear during exhalation, especially in patients with obstructive sleep apnea.

*Squawks*: These are relatively short adventitious inhalation sounds of a musical nature, occasionally found in patients with interstitial lung disorders [**1**] [**11**]. Acoustically, these waveforms may resemble short wheezes, though they are often preceded by a crackle. The duration of squawks may vary between 50 and 400ms. The basic underlying mechanisms probably differ from those of wheezes in obstructive lung diseases. Squawks usually occur from the middle to the end of the inhalation cycle in bronchial hyperresponsiveness and infectious pneumonia.

*Stridor*: This is a very low-frequency wheeze originating in the larynx or trachea [**5**] [**9**] [**11**]. It appears most frequently during inspiration. It can be audible at the level of the mouth, trachea and chest wall without even using a stethoscope. Stridor can appear in whooping cough and laryngeal or tracheal stenosis.

*Wheeze*: This is a continuous adventitious musical sound [**1**] [**9**] [**11**]. Acoustically, it is characterized by periodic waveforms with a dominant frequency usually over 100 Hz and lasting over 100ms, thus always including at least 10 successive vibrations. Wheezes are usually associated with an airway obstruction resulted from various causes. If the wheeze essentially contains a single frequency, it is classed as monophonic; polyphonic wheezes contain several frequencies. A wheeze can be located at the site of an anatomic obstruction or can be diffused in cases of asthma. Shaharum *et al*. [**12**] recommend placing the stethoscope over the trachea to determine its type and avoid listening to the signal across the chest, which acts as a low-pass filter, thus preserving more signal bandwidth.

*Pleural friction rub* or simply *pleural rub*: This is an audible signal present in cases of pleurisy or other diseases affecting the chest cavity [**1**] [**9**] [**11**]. Pleural rubs are non-polyphonic sounds that can be characterized by their duration, approximately 15ms. These sounds are caused by the pleural membranes rubbing together during respiration. They can be heard during either inspiration or expiration. They are low in pitch (below 350 Hz) and can be caused by pleurisy or a pleural tumor. They are described as similar to the sound of walking on snow.

**Visualization methods**

*Pneumo-phonogram*: This is a simultaneous, overlapped display of sound signal and airflow over time, acquired/recorded during respiration [**1**] [**5**] [**11**].

**Fig. 2:**
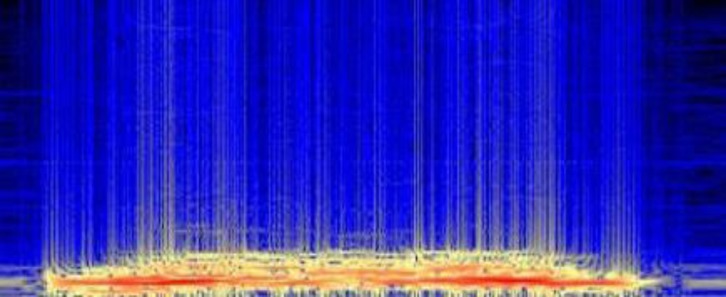
Example of a pneumo-spectrogram of normal breath sound (data collected in the ASAP project [Analysis of Auscultatory and Pathological Sounds] developed by the French National Agency for Research [ANR 2006 - TLOG 21 04]).

*Pneumo-spectrogram*: This is a tool displaying the time (on the abscissa), the frequency (on the ordinate) and the intensity of the signal (represented by a color palette) [**1**] [**5**] [**11**]. **Figure 2** gives an example of a spectrogram, showing the intensity ranging from black to blue, then yellow to red.

## Capture techniques

Clinicians use stethoscopes to help them diagnose respiratory disorders. However, here is a limited application of stethoscopes in research due to inherent inter-observer variability and subjectivity in the interpretation of respiratory sounds using this tool [**5**]. Improved diagnostic value of auscultation in detecting abnormal respiratory sounds in clinical research could be achieved by applying an objective and standardized approach when interpreting observations.

The computerized analysis of recorded respiratory sounds could offer a systematic approach to the diagnosis of different respiratory conditions *via* an automated classification of acoustic patterns. This process has the advantage of including an adapted capture chain of the sound preceding the analysis phase [**13**-**15**], typically consisting of the following elements [**3**] [**16**]: – Sound capturing: the position of the microphone is important, as the chest acts like a reducer and low-pass filter. Kraman *et al*. [**17**] studied the effects of different microphones and concluded that the most effective one was the electret microphone with conical coupler, 10-15mm in diameter; – Amplification of the signal; – Filtering and sampling; – Reduction of cardiac sounds and other noises produced by the skin during movement; – Sound recording.

Cheetham *et al.*[**18**] outlined the most significant points to consider when digitizing auscultation sound records, including sampling frequency, filtering, and signal-to-noise ratio introduced by the analog-to-digital conversion.

Considering these factors, it seems appropriate to mention “Egophony” [**19**] [**20**]. This is when a clinician asks the patient to pronounce the “E” vowel sound, while listening to their lungs with a stethoscope. Under normal conditions, the sound pronounced by the patient crosses the lungs and chest wall without any noticeable change. When the lungs are filled with liquid or a solid mass, such as tumor, however, the long “E” vowel sound is altered and sounds more like an “A”. This is known as the “E to A transition”, associated with fever, short breath and cough, indicating pneumonia.

**Acquisition**

Various methods and tools have been described to capture sound: – Using one microphone: This is the most frequently-used method. Usually, the sensor is an electret microphone. The most common sampling frequency used is that of telephony codecs (8k Hz), an analog-to-digital conversion with a 16-bit resolution [**1**] [**21**]. Another option is using an accelerometer, which is less sensitive to background noise [**1**] [**21**] but offers a much poorer performance compared to electret microphones. – Using several microphones and 3D representations. This technique makes it possible to identify where the sounds originate. It is a dynamic method that shows structural and functional properties, useful for diagnosis [**22**] [**23**]. – Emitting a sound then analyzing its propagation. This technique, described by Mazic et al. [**20**], consists of emitting a sound through a loudspeaker placed in the patient’s mouth. The method processes the characteristics of the signal’s propagation through the respiratory airways and chest, analyzing energy ratios, signal time delays and dominant frequency. – Closed-loop controlled ventilation measurement [**24**] [**25**].

In our studies [**5**] [**26**] [**27**], we focused on the method that uses one electret microphone.

**Noise filtering and cardiac-sound canceling**

Cardiac sounds can cause interference during lung sound analysis. The spectrum of heart sounds is 20-100 Hz. According to Elphick *et al.* [**28**], cardiac sounds can be attenuated using a simple band-pass filter [**50**-**2**, 500 Hz]. However, this filter must not be a high-pass filter (100 Hz) as the main components of lung sounds are also located in this frequency range. Consequently, several methods have been tested [**29**]: wavelets, adaptive filtering with a recursive least squares algorithm, time/frequency filtering, reconstruction, autoregressive/mobile average estimation (AR/MA) in time/frequency domain of wavelet coefficients, independent component analysis and the entropy-based method.

The filter method proposed by Bahoura *et al*. [**30**] is based on a wavelet packet transformation, using two filters that are defined in frequency and time. This filter method provides more accurate and effective results than its rivals, performing extremely well in experimental tests. Moreover, it enables better recognition of the characteristics of stationary signals (normal sounds or wheezes).

Yadollahi *et al*. [**31**] attempted to detect the different segments of sound that included cardiac sounds in order to suppress the cardiac component. They evaluated methods using Shannon’s entropy, Renyi’s entropy and multiresolution product of wavelet coefficients. The most efficient method was reported to be Shannon’s entropy.

Of all these methods, the best results were obtained with adaptive filtering [**32**], time/frequency filtering, and AR/MA estimation.

**Deleting parasitic noise**

When “cleaning” up respiratory sounds, it is also important to reduce background sound. There are two different methods able to achieve this [**3**]: – Noise reduction through adaptive filtering (deleting white Gaussian noise and vocal sounds, reducing measurement errors); – Noise reduction through wavelet packets (Donoho and Johnstone’s method)

More recent techniques involve simultaneous use of several sensors.

**Electrical safety**

There is no specific standard for electronic stethoscopes or recording devices, except that they need to conform to the European norm EN 60 601-1. Given that the device is applied to the skin of the patient, however, safety is critical.

In his study, Laszlo [**16**] included a reminder that the transducers themselves are passive, but the metallic part of the head needs to conform to the “low safety voltage” directive. The bias voltage is low and comes from a battery (the “low safety voltage” must be less than 60 V d.c.); the condenser, if present, must have very low capacity (20-100 pF). Thus, the only current that could reach the patient is a dispersion current coming from the amplifier, which does not occur when using an amplifier conforming to the IEC guideline (IEC 601-1).

## Respiratory sound characteristics

It is commonly admitted that respiratory sound frequencies are in the range of 50-2,500 Hz, and tracheal sounds can reach up to 4,000 Hz. This means we can define a sampling frequency at 8 kHz [**1**]. The spectrum of cardiac sounds falls between 20 and 100 Hz for basic signals and higher frequency (over 500 Hz) for breaths. Abnormal sounds can be divided into two sub-classes [**29**]: – Continuous or stationary sounds, *e.g.,* wheezes, rhonchus.; – Discontinuous or non-stationary sounds, *e.g.,* fine or coarse crackles.

Other adventitious sounds include squawk, snoring, and stridor. Below, we outline the characteristics of the two most extensively studied noises: wheezes and crackles [**1**]:

**Characteristics of respiratory cycles**

Based on his description of the analysis methods used, Bahoura [**3**] also proposed his definition of inspiration and expiration sound characteristics. He reported that tracheal sound frequencies are 60-600 Hz during inspiration and 60-700 Hz during expiration. He proposes using the Fourier transform with 4,096 points and two types of respiratory sound representations: – The waterfall method presenting the spectrum in three dimensions (amplitude, frequency, and time); – The spectrogram method described earlier in this article [**5**].

**Fig. 3:**
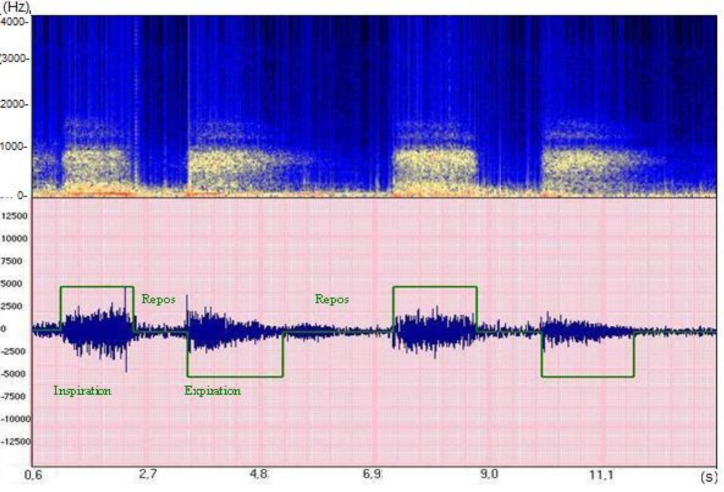
Breathing cycles. This represents the time and frequency characteristics of normal vesicular sounds. The upper green square represents the inspiration phase and the lower one the expiration. This example illustrates that there is relatively the same frequency range in both phases. Intensity decreases during expiration, mainly due to the loss in high pitches. In both phases, the frequency energy is distributed between some Hz and 1 kHz. The red line corresponds to a maximum of energy below 100 Hz (data collected in the ASAP project [Analysis of Auscultatory and Pathological Sounds] developed by the French National Agency for Research [ANR 2006 - TLOG 21 04]).

These representations generally enable a good visualization of respiratory cycles (**Fig. 3**).

**Normal respiratory sounds**

Various normal sounds can be heard depending on where the stethoscope is positioned [**1**] [**5**] [**9**] [**11**].

**Vesicular sounds**

Vesicular murmur can be heard on auscultation over most of the lung areas [**1**] [**5**] [**9**] [**11**]. These are quite clearly audible during inspiration, yet can only be heard during the beginning of the expiration phase. They are of a low, soft intensity that can be weakened in following circumstances: extensive thickening of the chest wall (for instance, in cases of obesity or emphysema). They are entirely absent in the following cases: – The lung is collapsed by the pressure of fluid or air in the pleural cavity, such as in cases of pneumothorax or pleurisy; – Absence of ventilation in the affected lung area, for example, in cases of lung compression, especially in atelectasis with retraction; – Following pneumonectomy, on the operated side [**1**] [**5**].

**Bronchovesicular sounds**

Normal bronchovesicular sounds can be heard between the scapulae at the posterior chest and center part of the anterior chest [**1**] [**9**] [**11**].

**Bronchial sounds**

Bronchial sounds are audible over the chest near the second and third intercostal spaces [**1**] [**5**] [**11**]. They are similar to tracheal sounds, high in pitch and can be heard during both inspiration and expiration. They are more clearly heard than vesicular sounds during expiration. The sounds are high-pitched (higher than vesicular sounds), loud and tubular.

**Tracheal sounds**

These can be heard over the trachea, above the sternum, in the suprasternal notch and fall in a frequency range of 100-4,000 Hz [**5**] [**9**]. They are generated by turbulent airflow passing through the pharynx and glottis. These sounds are not filtered by the chest wall and thus provide more information.

**Mouth sounds**

Mouth sounds are described as falling in a frequency range of 200-2,000 Hz. They represent turbulent airflow below the glottis [**5**]. In the case of a healthy person, there should be no sound coming from the mouth during respiration.

**Abnormal respiratory sounds**

Abnormal breath sounds include the absence or reduced intensity of sounds where they should be heard or, by contrast, the presence of sounds where there should be none, as well as the presence of adventitious sounds [**1**] [**5**] [**9**] [**11**]. Adventitious sounds are sounds that are superimposed over normal breath sounds. Adventitious sounds may be continuous or discontinuous based on their duration.

**Continuous adventitious sounds**

Continuous adventitious sounds (CAS) are characterized by a duration of over 250ms [**1**] [**5**] [**9**] [**11**]. They can be further subdivided based on their pitch: low-pitch CAS including rhonchi and squawks; high-pitched CAS including wheezes or stridor.

**Characteristics of wheezes and whistles**

Having bronchial origin and variable intensity, wheezes can be audible even at a distance from the patient [**5**] [**9**] [**11**]. They include wheezes heard during inspiration and sibilant wheezes heard during both phases.

Wheezes and rhonchi are CAS that can be heard in most cases during expiration and sometimes during inspiration or both phases. Wheezes are high-pitched, while rhonchi are low-pitched. Wheezes are caused by an airway narrowing which limits airflow, while rhonchus is the consequence of thickened mucus in the larger airways.

Wheezes, which are defined by Laennec as dry wheezing groans or wheezing, are sounds that last for longer than 50ms [**33**] or 100ms and less than 250ms [**34**].

The identification of continuous adventitious breath sounds, such as wheezes in the respiratory cycle, is extremely important for diagnosing airway obstruction pathologies [**34**]. Sovijarvi *et al*. [**1**]1 state that wheezes can present acoustically as symptoms of abnormalities in the respiratory system and can also indicate the severity and location of the most frequently-found airway obstructions in asthma and respiratory stenosis.

Localized wheezing can be heard during inspiration or both respiratory phases, at similar pitches, caused by a partial obstruction of the trachea or bronchi due to the presence of a tumor or foreign body.

Diffuse wheezing is most often bilateral, of various tonalities and particularly audible at the end of expiration, encountered in instances of bronchial asthma. In chronic obstructive bronchitis (bronchial pneumonia), diffuse expiratory wheezing can also manifest due to the vibration of the bronchial walls which tend to collapse during expiration.

Wheeze frequency falls within 100 and 2500 Hz, with a fundamental frequency between 100 (or 400 [**30**]) and 1,000 Hz [**33**] or 1600 Hz [**34**]. However, some authors have reported that wheezes have a dominant frequency greater than 400 Hz [**30**], contrary to rhonchus, whose dominant frequency is of 200 Hz and below. The spectrogram below shows that wheeze exhibits several harmonic frequencies, usually up to three.

**Fig. 4:**
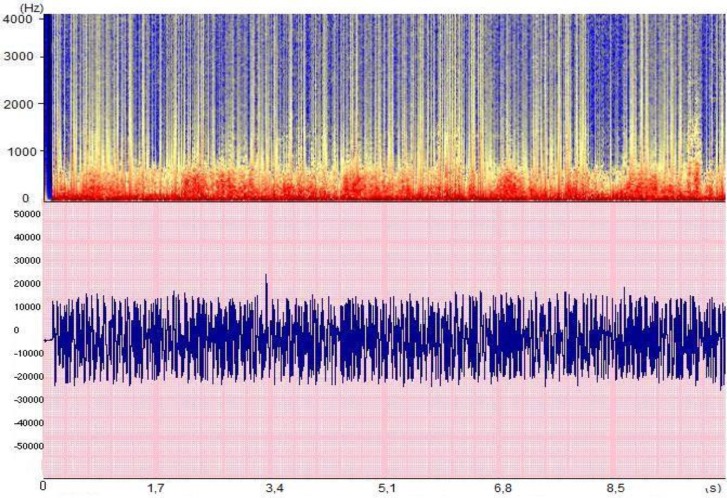
Example of a pneumo-phonogram and spectrogram of bronchiolitis with numerous wheezes in preschool children (data collected in the ASAP project [Analysis of Auscultatory and Pathological Sounds] developed by the French National Agency for Research [ANR 2006 - TLOG 21 04]).

The association between bronchiolitis and wheeze has been well known for over 50 years (**Fig. 4**), although the underlying factors that could explain this association are not yet understood. Infants aged <6 months are at high-risk of recurrent wheeze in early winter. Furthermore, infants with early and severe bronchiolitis requiring hospital admission are at significantly higher risk for both recurrent wheeze and subsequent asthma [**31**]. This long-known association [**32**] is not only well-documented in respiratory syncytial virus (RSV) bronchiolitis [**31**], but has also recently been linked with other viruses [**35**].

Identifying continuous adventitious sounds like wheezes in the breathing cycle is of great importance for detecting pathologies linked to airway obstruction. Sovijarvi *et al*. declared that wheezes demonstrate not only the presence but also the severity and location of the airway obstruction.

Asthmatic subjects typically wheeze during expiration, each wheeze manifesting over a duration of 80-250ms [**20**].

**Fig. 5:**
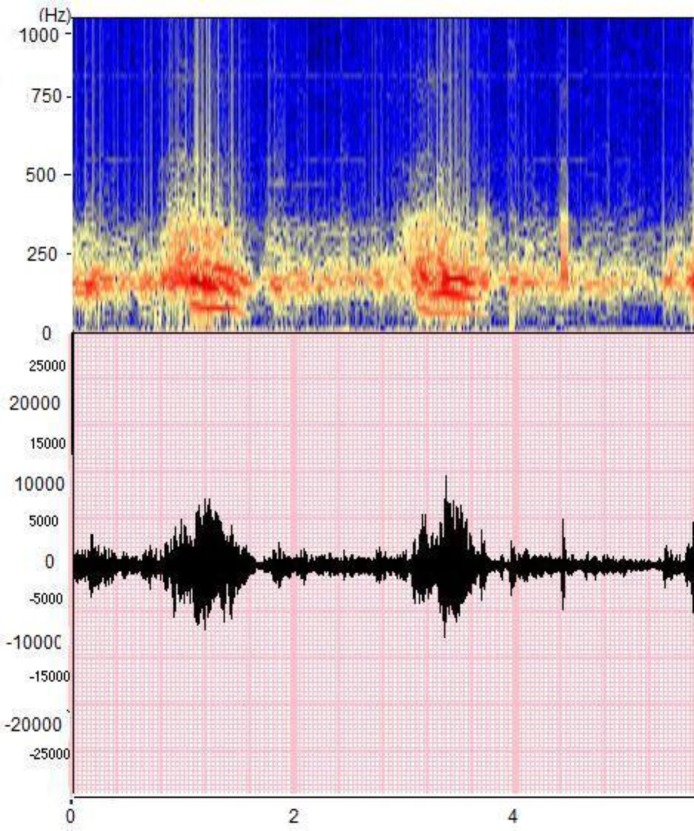
Example of a pneumo-phonogram and spectrogram of a wheezing sound in the context of asthma (data collected in the ASAP project [Analysis of Auscultatory and Pathological Sounds] developed by the French National Agency for Research [ANR 2006 - TLOG 21 04])

Fiz *et al*. [**36**] and Albers *et al*. [**37**] were able to objectively identify the presence of an obstructive pathology. Likewise, Meslier *et al.* [**38**] associate wheeze with the following pathologies: – Infections, such as croup (generally affecting infants under 3 years old), whooping cough, laryngitis, and acute trachea-bronchiolitis; – Laryngeal-, trachea-, or bronchomalacia; – Laryngeal or tracheal tumors; – Tracheal stenosis; – Emotional laryngeal stenosis; – Foreign body aspiration; – Airway compression; – Asthma (**Fig. 5**) [**39**], identification of physiological nocturnal wheeze [**40**]; – Chronic obstructive pulmonary disease; – Foreign body in the airways.

**Rhonchi or snoring**

Also of bronchial origin, sonorous rhonchi are low-pitched, occur during both inspiration and expiration and are altered by coughing [**9**] [**11**]. They are encountered in acute or chronic bronchitis accompanied by bronchial hypersecretion. They are usually cleared by coughing, except in cases of “fixed rhonchus” in which coughing does not dislodge them, generally associated with downstream foreign body airway obstruction.

**Characteristics of stridor**

**Fig. 6:**
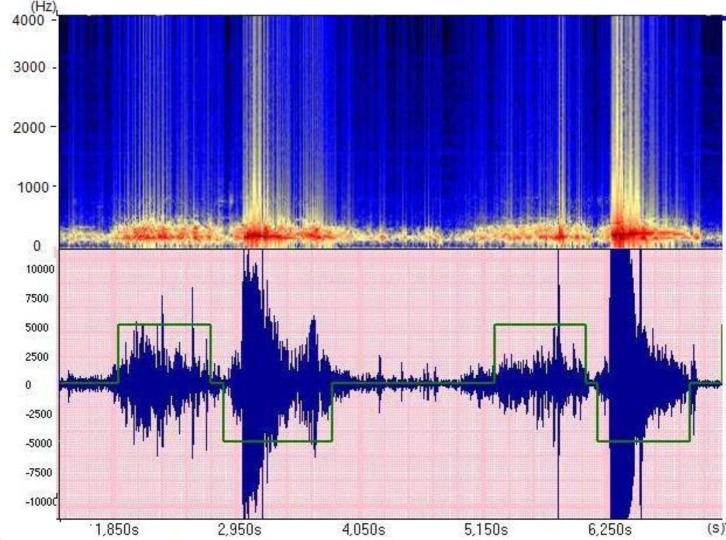
Example of a pneumo-phonogram and spectrogram of a stridor sound in the context of tracheal carcinoma (data collected in the ASAP project [Analysis of Auscultatory and Pathological Sounds] developed by the French National Agency for Research [ANR 2006 - TLOG 21 04]).

Inspiration requires much more energy than expiration [**5**] [**9**] typically below 500 Hz, but with some peaks at over 2,000 Hz (**Fig. 6**).

Stridor is a wheeze-like CAS of a sibilant and musical nature. It is primarily heard during inspiration. It is caused by a turbulent airflow in the larynx or lower bronchial tree. Associated diseases are epiglottitis, foreign body, croup or laryngeal edema. Stridor is high-pitched (above 500 Hz) and lasts for over 250ms.

**Squawk**

Squawks are continuous sounds lasting approximately 200ms [**1**] [**11**]. A squawk usually occurs during inspiration, is low-pitched (200 to 300 Hz), and often accompanies pneumonia.

**Discontinuous adventitious sounds (DAS)**

**Characteristics of crackles**

**Fig. 7:**
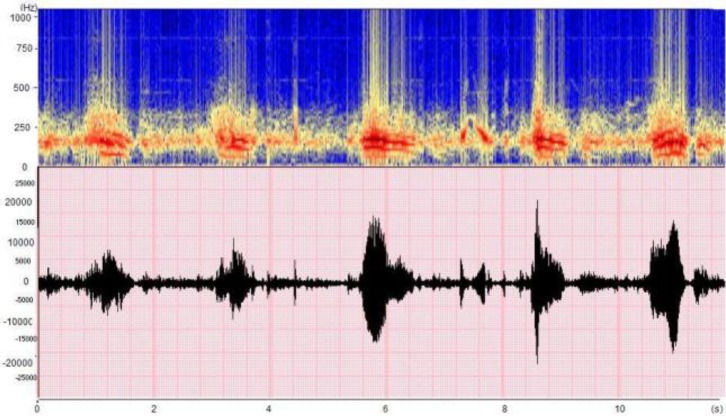
Example of a pneumo-phonogram and spectrogram of crackles in the context of pneumonia (data collected in the ASAP project [Analysis of Auscultatory and Pathological Sounds] developed by the French National Agency for Research [ANR 2006 - TLOG 21 04]).

Crackles are short explosive sounds, generally associated with pulmonary disorders [**41**-**43**] (for instance lung infection, pneumonia or pulmonary edema). They are typically generated when the airways that were abnormally closed during inspiration open up, or when the airways close at the end of expiration (**Fig. 7**).

Crackle detection is important because the number of generated cracks can potentially indicate the severity of a pulmonary disorder [**41**] or airway disorders [**44**]. Nevertheless, even more important than counting the crackles is locating them in the respiratory cycle and analyzing their waveform, both of which can inform physicians on the type of lung pathology [**1**].

**Fig. 8:**
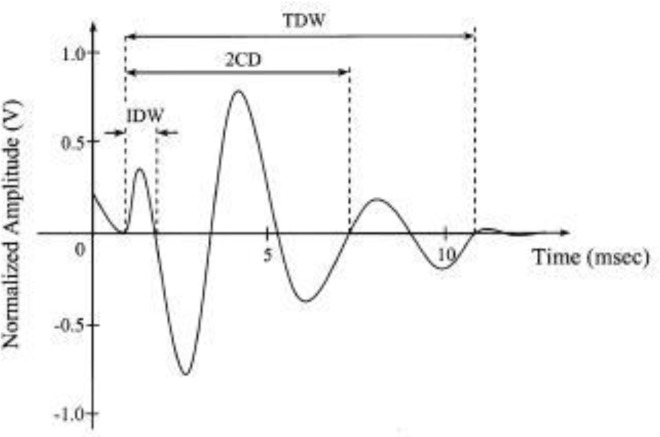
The waveform of a crackle

Crackles generally begin with a width deflection, followed by a long damped sinusoidal wave [**45**] [**46**], as represented in **Fig. 8**. Initial deflection width (IDW) represents the duration between the beginning of the crackle and the first deflection. Two-cycle duration (2CD) is the duration from the beginning of the crackle to the time at which the waveform completed two cycles. TDW corresponds to the total duration of the signal crackle. Crackles have been defined [**30**] as lasting for less than 20ms and ranging between 100 and 200 Hz in frequency.

In addition, crackles can be divided into two families: – Fine crackles (what Laennec labels “wet groan”) that are characterized ([**47**] and [**48**], respectively) as IDW=0.50ms or 0.90ms, 2CD=3.3ms or 6ms and TDW=4ms. These are exclusively detected during inspiration. Fine crackles are usually associated with pneumonia, congestive heart failure and lung fibrosis. Also called fine rales, they emit discontinuous, thin, dry noises that are high and evenly pitched, occurring in spells during inspiration. They become more distinctive after coughing and usually point to an alveolar disease process. Due to alveolar wall detachment and their pathological features, they are observed primarily in cases of pneumonia and interstitial alveolar or pulmonary edema subsequent to heart failure, though it can also occur in pulmonary fibrosis and some cases of interstitial pneumonia. – Coarse crackles are characterized by IDW=1.0ms, 2CD=5.1ms, TDW=6.7ms for [**43**] and by IDW=1.25ms, 2CD=9.50ms for [**46**]. They generally occur during inspiration, but can also be produced during expiration. Coarse crackles are generated by air bubbles in the large bronchi. They occur with chronic bronchitis and chronic obstructive pulmonary disease (COPD). Also called mucous rales and bubbling rales, coarse crackles or rales are discontinuous and of short duration. The sound emitted is irregular, uneven, intense, and observed in both phases of respiration, changing after coughing. They make a gurgling noise in the large airways and are often associated with bronchial congestion due to mucus hypersecretion. They are predominantly observed in bronchitis.

Piirilä *et al.* [**41**] described the principal pathologies where crackles can be found: – Pulmonary fibrosis (2CD <8ms, approximate frequency of 200 Hz); – Asbestosis (approximate duration of 10ms); – Bronchiectasis (2CD >9ms, generally appearing late in the inspiratory cycle and are relatively long compared to the respiratory phase); – COPD (2CD >9ms, generally starting early in inspiration and ending before the mid-point of inspiration); – Heart failure (2CD >10ms); – Pneumonia (2CD between 9 and 11ms, appearing at the mid-point of inspiration); – Sarcoidosis.

**Pleural rub**

A pleural rub is a discontinuous signal lasting over 15ms and pitched below 350 Hz [**1**]. This non-polyphonic (musical) signal is generated by the pleural membranes rubbing against each other. This may be caused by an inflammation of the lung membrane or a lung tumor. It can be heard during both inspiration and expiration.

Some authors describe a pleural rub as a dry grating and superficial sound, unchanged by coughing. It can be discrete in intensity, such as parchment rust, or louder, such as the creaking of new leather. It occurs at the onset of pleurisy, at its upper limit or after fluid evacuation. The differential diagnosis with coarse crackles may be challenging, although unlike the latter, the pleural rubs appear soon after the start of inspiration.

**Summary of normal and adventitious sounds**

**Normal breath sounds**

Pramono *et al*. [**49**] provide a useful summary of normal breath sound characteristics. As shown in **Table 1**, most of the energy is below 1,000 Hz for the signals captured over the chest. This increases to up to 2,000 Hz in the mouth and 5,000 Hz over the trachea, with a drop at 800 Hz.

**Table 1:** Normal breath sounds.

Breath sound	Location	Frequency range
**Vesicular**	Most of the lung	100 to 1,000 Hz, with energy drop at 200 Hz
**Broncho vesicular**	Between the scapula and center of the back	100 to 2,000 Hz, with energy drop at 600 Hz
**Bronchial**	The area between second and third intercostal spaces	100 to 4,000 Hz, with energy drop at 800 Hz
**Tracheal**	Over the trachea	100 to 4,000 Hz, with energy drop at 800 Hz
**Mouth**	In the mouth	200 to 2,000 Hz

**Summary of adventitious sounds**

**Table 2** presents the details of adventitious sound energy [**1**] [**11**].

**Table 2:** Adventitious sounds.

Sound	Duration	Phase	Frequency range	Disease
**Wheeze**	>80ms	BI and mostly BO	>400 Hz	Asthma, COPD, foreign body
**Rhonchus**	>80ms	BI and mostly BO	<200 Hz	Bronchitis, COPD
**Stridor**	>250ms	Mostly BI, BO, both	>500 Hz	Epiglottitis, foreign body, laryngeal edema
**Fine crackle**	~5ms	BI	650 Hz	Pneumonia, congestive heart failure, lung fibrosis
**Coarse crackle**	~15ms	Mostly BI, BO, both	350 Hz	Chronic bronchitis, bronchiectasis, COPD
**Pleural rub**	>15ms	BI and BO	<350 Hz	Inflammation of lung membrane, lung tumor
**Squawk**	~200ms	BI	200 to 300 Hz	Pneumonia

ms: milliseconds. BI: breathing in. BO: breathing out. COPD: chro nic obstructive pulmonary disease.

## Detection of known markers

The known markers commonly analyzed by automatic tools are crackles and wheezes. The principal algorithm families of detection of these markers are summarized in **Table 3**. Different analysis methods are described, including temporal analysis of the waveform for crackle detection, frequency analysis (Fourier transform, spectrogram in 2D or 3D [**1**] and sonogram [**50**] used for wheeze detection.

**Table 3:** Principal algorithm families of detection of the known markers.

Signal	Characteristics and processing [**7**]	Analysis
**Normal sounds**		
**Lungs**	Low-pass filtering (between 100 and 1,000 Hz)	Periodogram (power spectral density - PSD), autoregressive models [**38**]**Error! Reference source not found.**
**Trachea**	Noise with resonances [100, 3,000 Hz]	
**Adventitious sounds**		
**Wheezes**	Sinusoid (range ~ 100 and 1,000 Hz; duration >80ms)	Periodogram (PSD), STFT (short-time Fourier transform), FFT, linear prediction of coefficients [**69**], genetic algorithms [**27**], neural networks [**27**],, wavelet [**46**]
**Rhonchi**	Series of sinusoid ( <300 Hz and a duration >100ms)	
**Crackles**	Wave deflection (duration typically <20ms)	Temporal analysis [**44**], FFT, linear prediction of coefficients [**45**], fuzzy non stationary filter [**41**], genetic algorithms [**27**], neural networks [**27**], wavelet [**27**] [**46**]
**Snores**		Temporal analysis, periodogram (PSD)[**38**]
**Stridors**		Periodogram (PSD), STFT, autoregressive models [**27**] [**44**]

In techniques of spectral analysis, the main parameters are the average frequency of the spectrum, the frequency of maximal power, the number of dominant peaks, and the exponential decay. Finally, time-amplitude and time-frequency analyses are classically implemented using wavelet transform.

Among the different complex solutions, there are possibilities of using multi-layer perception in a neuronal network, genetic algorithms or a hybrid solution of both. The parameters search is performed using a learning method. Guler *et al*. [**51**] remarked that the hybrid solution is the most effective.

Finally, Murphy *et al.* [**52**] demonstrated that a multi-channel analyzer (several sensors used simultaneously) is able to detect significant differences between the pulmonary sounds of patients suffering from pneumonia and those of asymptomatic patients.

**Wheeze detection**

As explained above, Bahoura *et al.* describe a spectral analysis technique for wheeze detection. The main characteristic of all sounds is peaks of energy that can be visualized in the spectrum. The limitations of this method lie in the existence of peaks even in the normal pulmonary sounds resembling those that indicate wheezes, and consequently there is a significant rate of erroneous detections generated.

The difficulties encountered during automatic wheeze detection can be overcome using a joint time-frequency analysis [**5**]. The principle is as follows: detection in the frequency of a peak that could correspond to a wheeze is verified by a second time test in order to confirm true wheezes and reject the false ones.

According to Homs-Cobrera *et al*. [**53**], the significant parameters are frequencies and the mean number of wheezes detected. These authors used the following parameters: the number of wheezes, mean wheeze frequency with the highest power peak, mean wheeze frequency with the highest the mean power, mean frequency, and the percentile of maneuver occupied by wheezes. The parameters are defined by dividing the frequency range into 50 Hz bands from 150 to 200 Hz. Moreover, the present algorithm indicates that there is a significant correlation between the number of wheezes detected and the signal amplitude, due to a simultaneous dependence between the normalization factor and fuzzy rule thresholds. Spectrograms provide a graphical time-frequency representation of wheeze locations. Nevertheless, this is not sufficient to objectively characterize sounds.

Another process used for automatic wheeze detection was proposed by Bahoura and Homs-Cobrera et al. [**3**] [**53**]. This approach is based on the use of wavelet packet decomposition and consists of two stages. Firstly, frequencies are detected with wheeze extraction. Then, inverse transform and reconstruction of the useful signal are performed, followed by temporal detection, another step rendering it possible to eliminate false detection generated by superposition of the spectral domains of some normal sounds and wheezes.

Extrapolating from the spectrograms generated with recorded sounds, Lin *et al*. [**54**] made a 2D bilateral filtering process for edge-preserving smoothing. The results demonstrated the system’s high efficiency and the authors aim to use this system for asthmatic patient monitoring and the study of airway physiology. Similarly, a method of the continuous wavelet transform is described in [**34**], combined with a scale-dependent threshold. This method seems to provide a higher detection rate.

Meslier and Charbonneau’s research [**38**] also describes the automatic wheeze analysis and the quantification of spectral analysis. These algorithms are based on the definition of a threshold upon which the presence of peaks in frequency is characteristic of a wheeze. This threshold differs among the different articles, with a peak at times characterized by a 15 times greater power than the current average or 3 times greater than the average value. All these studies define constant thresholds based on power measurements.

Qui *et al*. [**55**] confirmed that frequency analysis alone generates a relatively significant number of erroneous detections. Their paper describes a new algorithm based on auditory modeling called “frequency and duration dependent threshold (fddt) algorithm”. In their approach, the parameters for average frequency and wheeze duration are obtained automatically and the notion of threshold depends on the frequency and duration introduced in a new wheeze detection algorithm. The threshold is no longer based on global power, but rather on power corresponding to a particular frequency range.

The choice of energy instead of power was made based on previous study results, and the latter indicates that an energy threshold is more suited to short-duration sound detection (lower than 200ms).

**Crackle detection**

Crackle analysis can be divided into three major stages: – Application of a noise reduction filter in order to delete the residual stationary noise in a non-stationary signal; – Search for the waveform corresponding to a crackle; – Classification of detected crackles into two categories: fine and coarse.

Kayha and Yilmaz [**56**] proposed an automatic system of crackle detection and classification. They used a stationary/non-stationary filter and a wavelet packet transform (also called WPST-NST) that enables crackles to be isolated from vesicular sounds.

Kawamura *et al.* [**57**] demonstrated the existence of a correlation between respiratory sounds and high-resolution computed tomography findings. Two parameters, 2CD and the IDW of crackles, were induced by time-expanded waveform analysis.

Kayha *et al*. [**58**] describe a system based on increasing transient to background ratio by adaptive filtering and implementing nonlinear operators to wavelet decomposed lung sounds.

Yeginerand *et al*. also describe in their paper [**45**] the utilization of wavelet networks in order to model pulmonary crackles.

The algorithm proposed by Vannuccini *et al*. [**46**] uses a stationary/non-stationary fuzzy-based filter (FST-NST). The results of the separation demonstrated relatively good accuracy. Their proposed algorithm deals with non-stationary crackles and fuzzy rules. The FST-NST filter was applied to sounds coming from three databases. First, crackles were separated from the vesicular sounds. Next, fuzzy “if-then” rules were used. The results of the separation are reliable, objective and of high quality, as the FST-NST filter was found able to automatically identify the location of crackles in the original signal.

Hadjileontiadis *et al*. [**46**] detected crackles and bowel sounds using a fractal dimension analysis of the records. Their results seem to be conclusive and, moreover, their method robust to noise stress.

Our comparison of the results of these different methods is summarized in **Table 4**. The most fitting classifying results were obtained using wavelet analysis. The representation of Prony’s method indicates a correlation between the type of pathology, crackle occurrence compared to pulmonary volume, and Prony’s frequency [**56**].

**Table 4:** Summary of several methods of crackle detection.

Methodology	Parameters	References
**Time-frequency analysis**	Gaussian band width, peak frequency, total deflection width, maximal deflection width	[**46**] (correct classification level: 87.78%)
**Time-frequency analysis**	Gaussian band width, peak frequency, maximal deflection width	[**46**] (correct classification level: 90.5% )
**Prony modeling**	Parameters of the Prony model	[**46**] [**27**] (correct classification level: 63.89%)
**Wavelet transform**	Autoregressive coefficients	[**1**] [**64**]
	Wavelet scale	[**46**] [**27**] (correct classification level: 93.9%)
	Wavelet transform fractal dimension based	[**14**] [**46**]
	Wavelet transform stationary – non stationary	[**27**]
**Fuzzy rule-based system – FST-NST**	27 fuzzy rules	[**46**]
**Artificial neural networks**	Autoregressive coefficients, wavelet coefficients, crackle parameters	[**46**] [**27**]
**Empirical mode decomposition**	Intrinsic mode function: local zero mean oscillating waves obtained by sifting process	[**27**]

FST-NST: stationary-non-stationary fuzzy-based filter.

**Respiratory cycle detection**

In order to provide exploitable results, information must always be considered in the context of a respiratory cycle [**28**] It is thus useful to automatically detect inspiration/expiration phases. Benedetto *et al.* described another characteristic of the pulmonary signal: the spectral power of pulmonary sounds during inspiration is higher than those during expiration [**24**]. This characteristic can be used alone to enable stage detection. Likewise, Chuah and Moussavi [**4**] processed the average value of the spectral power to qualify the respiratory cycle. Their analysis was completed by processing the average value of tracheal spectral power to determine the beginning of respiration.

Moussari *et al*. [**59**] used the average power spectrum of the breath signal and the difference between the average tracheal power spectrum and chest signal to detect the respiratory phase. Their results accurately classified the phase between 31 and 69% of the time. Also, the average power spectrum difference between inspiration and expiration in a frequency range of 150-450 Hz was maximum 10 dB. While this method is adequate for artificial sounds, however, it does not enable real auscultation sounds to be classified. Finally, Leng Y *et al.* proposed measuring sound while using a fractal dimension and a parameter called “variance fractal dimension” [**60**].

Contrary to crackle or wheeze detection, the main methods of respiratory phase detection use artificial intelligence algorithms. Guler *et al*. [**61**], for example, used a six-phase classification: beginning, middle and end inspiration, along with beginning, middle, and end expiration. This method depends on the use of multistage classification. The extracted features are autoregressive parameters and cepstral coefficients.

The development of this type of tool is subject to two major difficulties: – Respiratory signals are not stationary because lung volume constantly changes; – Respiratory sounds present great variability depending on age, mass, and pathology.

Guler *et al*. based their study on a multilayer perceptron (MLP) [**51**]. On individual segments, it provides approximately 60% accurate recognition in the expert phase.

Carlos and Verbandt [**62**] used two artificial independent neural networks (ANN): their algorithm is based on two neural networks: inspiration ANN and expiration ANN. First, pre-processing is performed, which normalizes the signal in amplitude (between 0 and 1). The next stage consists of the ANN with one hidden layer. The parameters are obtained by means of a learning algorithm using a back-propagation technique. Following this, post-processing is applied, consisting in removing the uncertain “1” situated between at least five “0” and inversely.

## Other auscultation methods

**Coin test**

This test is particularly useful in cases of pneumothorax, large bulla, and hydro-pneumothorax [**1**] [**11**]. With patients sitting or standing, a metallic coin is placed flat against the chest just below the mid-clavicle and struck with the edge of another coin with the help of an assistant. The stethoscope diaphragm is positioned to listen at the same point on back of the chest. The coin test is positive if high-pitched, metallic, bell-like sounds are heard.

**Scratch sign test**

This test is rarely used, yet also useful for diagnosing pneumothorax [**1**] [**11**]. The patient is sitting or supine. The physician places the stethoscope diaphragm to listen at the midpoint of the sternum and surface of the chest wall and equidistant points on the left and right of the instrument are scratched with the fingers. When the site of pneumothorax is scratched, the sound heard is louder.

**The “99” test**

Many have experienced this without necessarily knowing the rationale behind it. During the doctor’s auscultation, the patient is asked to say “99” (or “33” if you are in France) [**1**] [**5**]. This is not at all to check the patient’s pronunciation! Nor is it to make any kind of calculation; it is for a medical purpose. When this number is said, the low-pitched sound causes vibrations at the level of the larynx. Using a stethoscope, the physician listens to the air flowing in the lungs at the same time. The waves generated by saying “99” propagate all throughout the chest, differing only in areas affected by pneumothorax (sound is absent) or atelectasis (compact area where sound is better transmitted).

**The seismic test**

The name of this test comes from the tests performed to detect the presence of petroleum. Vibrating waves are propagated through the mouth of the patient [**1**] [**11**] while the doctor listens to the sound emanating from the chest. Distortion of the sound is linked to the amount of water crossed by the audible wave.

**Percussion**

With the patient sitting or standing, the practitioner taps on the front of the chest and listens at the back with a stethoscope. The emitted note is louder in cases of pleural effusion.

## Respiratory sound classification

In lung medicine, there is no universal pattern or parameter threshold indicating the presence or absence of a pathology. Therefore, Zheng *et al*. [**63**] proposed the establishment of a personalized pattern, combining sound information and other patient measurements. They sought to recognize a pattern of pulmonary sounds. The method applied can be divided into two stages: characterizing the variables that can be extracted from the waveform of pulmonary sound, and recognizing the changes in these variables that will provide information concerning pattern variations.

Guler *et al*. [**61**] focused on artificial intelligence techniques. They combined a neural network and genetic algorithm to analyze respiratory sounds. First, they selected complete respiratory cycles, on which a PSD (power spectrum density) of 256 was applied. Then, an MLP neural network was employed in order to detect the presence or absence of adventitious sounds (wheezes and crackles). The search for optimal parameters was performed using a learning method. Each sound was associated with several characteristics and a diagnosis. A total of 129 specific characteristics were verified (PSD0 - PSD128). Following this, different learning rules were used in order to associate characteristics and diagnosis.

Kahya *et al*. made a comparison between k-NN (*k*-nearest neighbor) and ANNs. They used different features extracted from the respiratory signal, with each cycle divided into six segments comprising three features: autoregressive coefficient, wavelet coefficient and crackle parameters [**64**].

Moreover, the performance of the classifiers was measured using the following statistical parameters: – Sensitivity: the number of pathological subjects correctly classified /total number of pathological subjects; – Specificity: the number of healthy subjects correctly classified /total number of healthy subjects; – Accuracy: the number of subjects correctly classified/total number of subjects.

Then, they added crackle parameters to the observed features in order to increase the performance of classification [**65**]. As before, K-NN and multinomial classifiers were used. They observed that adding crackle parameters to feature vectors and fusing phase decisions improved classification results.

The study by Yeginer *et al*. [**66**] focuses on four pathologies: asthma, bronchiectasis, COPD, and pneumonia. The sound is divided into six subphases: early (30%), mid (40%) and late (30%) of both inspiration and expiration. Classification experiments were applied to each subphase. Neural classifiers (MLP with a hidden layer with ten nodes) were used with the following parameters: autoregressive parameters, error prediction, and expiration/inspiration duration ratio. The weight and biases of the MLP were updated using the Levenberg-Marquardt optimization algorithm, which is one of the fastest. After that, they classified the results in three stages: healthy/pathological classification, restrictive/obstructive classification, and pathology-specific classification (*e.g.,* asthma and bronchiectasis). The classification accuracy was calculated by the “global number of segments correctly classified/global number of segments”, recorded as approximately 70-80%.

The study by Pittner and Kamarthi [**67**] sought to describe a preprocessing method to reduce the entry pattern size in neural networks and increase the performance of estimation or classification. The results indicate that wavelet expansions are significant signal sensors and allow important features to be extracted.

Several authors have performed the classification of normal and adventitious sounds in two stages: linear prediction of coefficients (LCP) and features of the energy envelope [**68**] [**69**]. Seven types of respiratory sounds were thus classified, including the following four normal sounds: vesicular breath sounds (V), bronchial breath sounds (B), bronchovesicular breath sounds (BV) and tracheal breath sounds (T). The extracted features were: FFT, PDS estimation by means of LCP. Nevertheless, in this study, a manual decision of the inspiration/expiration periods was realized. The primary objectives were: to quantitatively characterize several respiratory sounds and to provide an automatic classification method of these types of sounds. Finally, the diagnosis was established by a physician, based on the sound analysis associated with other diagnostic values. Out of their 105 experiments, only five generated errors.

Dokur and Olmez [**70**] used wavelet transform, selecting the best samples for analysis by dynamic programming, then using a Grow and Learn neural network for classification. The decision process was made up of three stages: process normalization, feature extraction, and artificial neural network by classification.

MLP is frequently used in biomedical signal processing, yet it does present three main drawbacks: – Backpropagation algorithms take too long during the learning phase; – The number of nodes in the hidden layers must be defined before the learning phase. The structure is not automatically determined by the training algorithm; – Backpropagation algorithms may be impacted by local minima, decreasing network performances.

## Factors influencing the measurement

Several factors can interfere with auscultation signal analysis [**3**], modifying results and rendering the comparison between research centers more difficult [**71**]: – Age and corpulence of the patient; – Air volume changing in the lungs; – Location of sound capturing; – Breathing flow; – Position of the patient; – Characteristics of the measurement equipment.

Environmental conditions also have an influence on the results, since they can generate artifacts and bias. These will be discussed below.

**Age and corpulence**

Differences due to age are the most visible in infants. Elphick *et al*. [**50**] noted that stethoscope evaluation is not very accurate for wheeze and crackle detection in this population [**28**], and audible respiratory sounds in early childhood are known to present acoustic characteristics distinct from those generally heard in adults.

Therefore, Mazic *et al*. [**20**] proposed using more objective methods to automatically detect wheeze in asthmatic infants during forced breathing.

**Subject conditions**

For testing lung auscultation, several academic societies and authors have published guidelines that are helpful, even for short-term recording [**5**] [**72**]. These include body position, the location of the microphones and respiratory maneuvers.

Body position: it is recommended that the patient is sitting down, with hands on the thighs to avoid the arms interfering with the axillary areas where the microphones are located.

As a large number of sites were adopted (>50) by the investigators, there is a need to anatomically define the microphone locations that have proved to be most relevant: trachea (on the sternal notch); chest (posterior, anterior, lateral). Sovijarvi *et al*. provide a list of standard positions [**10**] [**12**] [**14**].

**Non-stationary signals linked to lung air volume variations**

The static characterization of the process evolves during the respiratory cycle [**1**] [**39**]. Respiratory sounds are non-stationary, particularly due to the changing lung volume [**1**] [**72**]. Thus, in order to correctly interpret the results, it is recommended to bring back the pulmonary air volume.

**Respiratory maneuvers**

The airflow and volume have a strong influence on the collected sounds [**72**]. In particular, wheeze and crackle characteristics and amount are related to the air volume as well as the disease. For instance, crackles have been shown to appear early in inspiration in patients with COPD and late in inspiration in patients with fibrosing alveolitis. Recording when crackles occur during the breathing cycles and calculating the number and position of crackles is thus helpful for establishing a diagnosis.

This can only be achieved with the help of automatic tools [**1**]. The automatic tool that we developed during the “*Analysis of Auscultatory and Pathological Sounds*” (ASAP) project, developed by the French National Agency for Research (ANR 2006 - TLOG 21 04) [**27**], includes wheeze and crackle detection, as well as positioning them during the breathing cycle.

**Environmental conditions**

Noise not related to the relevant breathing signal is generated by the body of the patient or by external sources. Standardization of noise conditions is addressed in references [**1**] [**9**] [**72**].

External sources can produce continuous and transient noise [**27**]. Continuous noises are those generated for example by motors, hard disks, air-conditioning ducts, fluorescent light bulbs, transformers, fans in electronic equipment and computers. These noises usually generate 50 Hz or 60 Hz harmonics and can therefore be very easily filtered. Traffic in the street is also a continuous noise, yet it does not produce harmonics. Transient noises include speech, music, noise from airplanes, trains, cars, slamming doors, furniture squeaks, phone ringtones, and alarms from monitors or other electronic devices.

Body sounds can either be those related to breathing (chest motion, muscle sounds, skin friction, sounds induced by airflow in devices, and tubes and valves for monitoring of airflow and volume) or not (heart sounds and murmurs, vascular sounds, swallowing, burping, bowel sounds, joint crackles, speech or other noises made by the patient) [**5**] [**27**].

External sounds can easily be filtered with the use of an additional sound capture that will record and subtract environmental noise. A project has even been proposed by the US army using auscultation inside helicopters, filtering external noise. However, the noise generated by the body itself cannot be detected by an additional microphone.

**Standardization of the measurement protocol**

In order to overcome these limitations, *The European Community* financed a BIOMED 1 Concerted Action project entitled “*Computerized Respiratory Sound Analysis*” (CORSA) that proposed to define a semiology adapted to collect and analyze respiratory sounds. These works concluded with a proposition of standardization that was proposed in the European project CORSA [**33**]. CORSA described auscultation points, type of sensors, filtering, sampling frequency, FFT technique, definition of a spectrogram average and use of standard flows.

## Automated respiratory sound analysis devices

Pramono *et al*. [**49**] provided a short list of available automated respiratory sound devices. These tools aim to overcome the limitations of conventional auscultation, which cannot provide continuous monitoring and needs to be performed by a specialist with prolonged experience, also involving the well-known limitations of the human ear that increase with ageing and are thus antonymic with experience [**5**] [**27**]. In addition, noises might disturb the auscultation process. Automatic tools are expected to overcome these drawbacks.

The *National Institute for Health Research* provides a list of already available commercial systems [**73**], such as: the *Wheezometer* [**74**], the *Wholter* [**75**], the *VRI* [**76**], the *LSA-2000* [**77**], the *LEOSound* [**78**], and the multichannel *STG* [**79**] and STG for PC [**80**] or handheld STG [**81**]. The *Wheezometer* and *Wholter* were developed by *Karmelsonix*^®^. The *Wheezometer* uses a sensor that needs to be placed over the trachea to detect wheezes and evaluates the percentage of time over which wheezes were detected in the breathing signal [**74**]. The *Wholter* is intended for home monitoring [**75**]. The *LSA-2000* has up to four sensors to attach on the chest to detect interstitial pneumonia [**77**]. The *LEOSound* uses three sensors to collect, analyze and store data for wheeze and cough detection [**78**]. *STG* tools were developed by Dr R. Murphy [**80**] [**81**]. The multichannel *STG* has 14 sensors placed at the level of the lungs, heart and trachea. It aims to identify wheezes, crackles, and rhonchi. Smaller devices have also been developed to be connected either to a PC or a handheld device. The above-mentioned devices do not yet have portable, easy-to-use versions.

## Conclusions/Future works

We are currently testing and studying different algorithms in the ASAP project [**5**] [**27**]. The next stage will consist in exploiting all the richness of the sound. This augmentation of the spectrum studied, linking it to signal analysis techniques, will enable the definition of new characteristic markers. Previous studies have demonstrated the need for an exhaustive scientific approach that accounts for both the definition of a semiology, the consolidation of definitions of known characteristics markers, the definition of common or even universal semantics, and the development of determining tools that enable the detection of these markers [**1**] [**5**] [**11**] [**27**]. This was precisely the aim of an ambitious study commenced in collaboration with LaenneXT^®^ in the “ASAP” project. This study is conducted by multidisciplinary teams, including medical experts from the University Hospital of Strasbourg (Strasbourg, France), “*Institut Régional des Cancers de l’Appareil Digestif*” (IRCAD) (https: / / fr.wikipedia.org / wiki / Institut_de_recherche_contre_les_cancers_de_l%27appareil_digestif), with support from the *Alcatel-Lucent*^®^ research teams [**27**]. Among the best-identified outcomes of the project, its strength lies in creating an auscultation school within the University of Strasbourg (Strasbourg, France) [**5**].

In this context, a recent meta-analysis from Gurung *et al*. [82] demonstrates that computerized analysis of recorded respiratory sounds could be a promising adjunct to chest auscultation as a diagnostic aid in both clinical and research settings. In this meta-analysis, Gurung *et al*. [82] found that computerized respiratory sound analysis offered a relatively high level of sensitivity and specificity in a small number of studies. The overall sensitivity for the detection of abnormal respiratory sounds using computerized lung sound analysis was 80% and overall specificity was 85%.

Acknowledgments: This work was performed within the framework of the projects from the MERCURE platform, and more specifically the STETAU and ASAP projects. We would like to acknowledge our partners on this project: CHRU Strasbourg, IRCAD, and Alcatel-Lucent Enterprise.
